# A107 VISCERAL ADIPOSE TISSUE VOLUME DIFFERENTIATES BETWEEN FIBROSTENOTIC AND INFLAMMATORY CROHN’S DISEASE

**DOI:** 10.1093/jcag/gwac036.107

**Published:** 2023-03-07

**Authors:** R Rosentreter, E Cheng, H Shen, C Ma, D Bhayana, R Panaccione, M Raman, A Medellin, C Lu

**Affiliations:** 1 Department of Medicine; 2 Department of Radiology; 3 Department of Mathematics and Statistics; 4 Department of Community Health Sciences, University of Calgary, Calgary, Canada

## Abstract

**Background:**

Creeping fat, a form of visceral adipose tissue (VAT) that wraps the intestinal wall, influences the formation of Crohn’s disease (CD) strictures. The degree of fat wrapping from intestinal stricture resections is correlated with the extent of chronic inflammation, fibrosis, stricture formation, and response to biologic therapy. VAT and subcutaneous adipose tissue (SAT) ratios from CTE (computed tomography) scans are elevated in CD strictures. However, the definition of strictures in these studies has been poorly defined and not included current well-recognized criteria: 1) bowel wall thickness (BWT), 2) narrowed luminal diameter, and 3) pre-stenotic dilation. (PSD).

**Purpose:**

The objective of this pilot study was to assess the relationship of 2D and 3D VAT:SAT ratios with CT stricture parameters in patients with terminal ileal (TI) CD strictures.

**Method:**

2D VAT:SAT ratios from CT’s of CD patients with TI strictures defined as increased BWT, narrowed luminal diameter (< 50% relative to normal adjacent distended loop), and PSD greater than the stricture diameter were retrospectively obtained from a database and chart review. CT’s from fibrostenotic CD patients were sex and BMI matched to patients with only TI inflammatory behaviour. Patient demographics, medication, smoking, and surgical history were also obtained. Analyses were adjusted for age, sex, and BMI covariates. Unpaired t-tests and multi-variable logistic regression analyses were conducted.

**Result(s):**

Twenty-eight patients with stricturing CD had a significantly greater mean VAT:SAT volume ratio than 29 non-stricturing CD (41.5 cm^3^ vs 34.2 cm^3^, p=0.03). Thirty-six percent (10/28) of CD stricture patients had prior ileocolic resection with a mean disease duration of 13.5 years (range 0-48). The median ileal BWT (7.0 mm, range 4.0-13.0 mm) for the stricturing group was significantly greater than those with inflammatory behaviour (BWT 2.0 mm, p<0.0001). The median luminal diameter and PSD for the stricture group was 2.0 mm (range 0 - 14.0 mm), and 3.0 cm (range 1.0 - 7.3 cm), respectively.

**Image:**

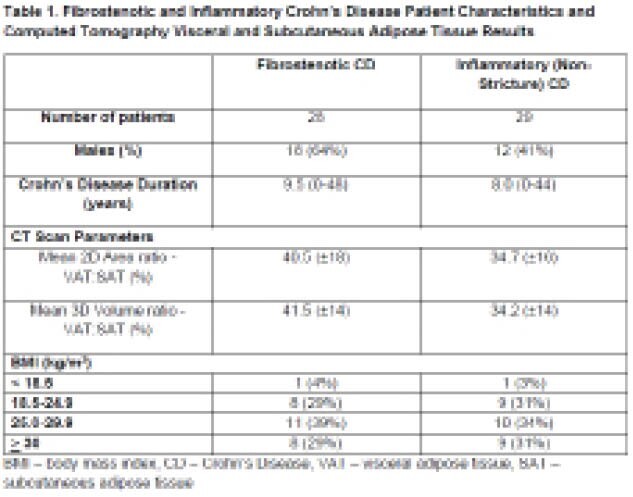

**Conclusion(s):**

Fibrostenotic TI CD patients have increased VAT:SAT ratios in comparison to those with only inflammatory behaviour. These pilot VAT:SAT results provide an initial foundation for further studies to assess its predictive role in responsiveness of medical or surgical therapies in stricturing CD.

**Please acknowledge all funding agencies by checking the applicable boxes below:**

None

**Disclosure of Interest:**

None Declared

